# Updated Review of the Management of and Guidelines for Traumatic Brain Injury

**DOI:** 10.3390/jcm14196796

**Published:** 2025-09-25

**Authors:** Aaron Rapp, Hassan Kobeissi, Daniel K. Fahim

**Affiliations:** 1 Department of Neurosurgery, Oakland University William Beaumont School of Medicine, Rochester, MI 48309, USA; 2 Department of Neurosurgery, Corewell Health William Beaumont University Hospital, Royal Oak, MI 48073, USA

**Keywords:** traumatic brain injury, intracranial pressure, decompressive craniectomy, clinical guidelines, RESCUEicp, DECRA

## Abstract

Traumatic brain injury (TBI) remains a leading cause of mortality and disability globally, necessitating ongoing research into its pathophysiology and management. This review compiles current knowledge on TBI, focusing on its mechanisms, updated clinical guidelines, and recent clinical trials. A systematic PubMed search identified studies on TBI management, guidelines, and trials from 2015 to 2024. TBI initiates with a primary mechanical insult, followed by secondary injury from cerebral edema, elevated intracranial pressure (ICP), and ischemia. Management hinges on stabilizing patients, mitigating secondary injury, and optimizing recovery, guided by the Brain Trauma Foundation’s 2020 guidelines. Key trials, including Trial of Decompressive Craniectomy for Traumatic Intracranial Hypertension (RESCUEicp) and Decompressive Craniectomy in Diffuse Traumatic Brain Injury (DECRA), have refined recommendations for decompressive craniectomy, though its role remains debated. Findings indicate that large craniectomies improve mortality in late refractory ICP cases, but early intervention lacks clear benefits. Challenges include access to advanced monitoring and surgical expertise. This review underscores the evolving nature of TBI care and the need for dynamic guidelines to improve patient outcomes.

## 1. Introduction

Traumatic brain injury (TBI) is a complex condition and a leading cause of death and disability worldwide, despite advances in modern medicine [1]. Defined as an alteration in brain function or evidence of brain pathology resulting from an external force, TBI ranges from mild concussions to severe injuries requiring prolonged interventions and rehabilitation [2]. Its profound impact on individuals, families, and healthcare systems highlights the need for a comprehensive understanding of its mechanisms and management. In 2021, TBI caused 69,000 deaths in the United States, with over 214,000 hospital admissions annually, primarily due to falls and firearm-related suicides [3]. Men are twice as likely to be affected, and 32% of admissions involve patients over the age of 75 [3].

Historically, TBI was thought to predominantly affect younger patients. The IMPACT studies, published in 2007, reported that the median age of patients was 30 years [4]. More recently, updated studies have suggested that TBI affects older cohorts. In the CENTER-TBI study, published in 2015, the median age of patients with TBI requiring hospitalization in an intensive care unit was 49 years, and 20% of patients were over 65 years [5]. Recognizing the volume of older patients required adjustments to the guidelines to consider the management variations for various aged patients who might not be able to tolerate as aggressive treatment as young and healthy trauma patients.

Patients who are hospitalized in the intensive care unit (ICU) with TBI frequently have concomitant extracranial injuries. A recent study reported that patients with TBI in the ICU had injuries involving the thorax, spine, and extremities [6]. These extracranial injuries contribute to the poor outcomes of patients with severe TBI. In addition to the multisystem injuries that patients with TBI experience, they often have systemic complications from their injury, including respiratory failure, renal failure, adrenal insufficiency, and myocardial injury. Due to the diverse patient population affected by TBI and systemic complications from TBI, management is complex.

Management of TBI consists of monitoring, medical treatment, and surgical intervention. The optimal management of patients with TBI is well studied; however, there is ongoing debate regarding optimal management strategies due to the complexity of the condition and variability in the severity of TBI. The Congress of Neurological Surgeons and the Brain Trauma Foundation publish guidelines detailing the management of TBI, with different levels of evidence to support their recommendations [7].

Landmark studies have shaped TBI management, sparking debates over aggressive surgical versus medical approaches. The Brain Trauma Foundation’s fourth-edition guidelines, updated in 2020 following the Trial of Decompressive Craniectomy for Traumatic Intracranial Hypertension (RESCUEicp) trial, reflect the rapid evolution of evidence [7]. Particularly, the RESCUEicp study demonstrated that decompressive craniectomy for patients with severe TBI resulted in decreased mortality compared with medical management alone. Furthermore, the trial also demonstrated that in the craniectomy group, although there were more survivors of severe TBI, their functional outcomes were poorer than those managed medically. This review explores TBI pathophysiology, compiles current clinical guidelines, outlines recent management strategies, and evaluates pivotal clinical trials from the past decade, reflecting on the state of TBI care as of April 2025.

## 2. Materials and Methods

### 2.1. Search Strategy and Eligibility Criteria

**Figure 1:**
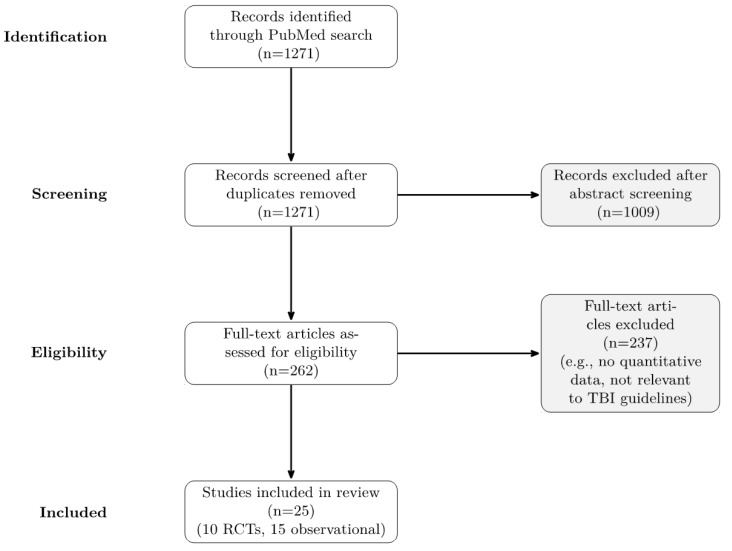
Flow chart detailing the literature review process.

A systematic PubMed search was conducted using the keywords “traumatic brain injury”, “guidelines”, and “management” for studies published between 1 January 2015 and 2024. The parameters initially yielded 1271 papers for review. Abstracts were then reviewed for initial consideration. Studies were included if they addressed recent updates to TBI management, guidelines, or clinical trials. Articles were excluded if they lacked quantitative data. The selection for inclusion is represented in Figure 1. It was not feasible to include data published in a language other than English because the authors’ institution is a United States-based medical school. The authors would be unable to accurately evaluate and review studies not published in English. Management strategies were compared against the Brain Trauma Foundation’s 2020 guidelines to create a comprehensive review. Major trials, including RESCUEicp and DECRA, were analyzed for their impact on guideline updates. The Congress of Neurological Surgeons’ shift to “living guidelines” was noted, allowing real-time updates to reflect new evidence without requiring frequent full guideline revisions [7]. The authors did not use artificial intelligence in the preparation of this publication.

### 2.2. Screening Process and Data Extraction

We included all eligible studies that fulfilled our inclusion and exclusion criteria. Two authors participated in the abstract and full-text screening process to ensure accuracy, and a third senior author was consulted to reach consensus. Extracted data included study characteristics, patient and demographic data of the included studies, and outcomes of interest. Two authors completed data extraction, and a third senior author was consulted in cases of discrepancies.

## 3. Results

### 3.1. Literature Search Results

The PubMed search identified 25 studies meeting inclusion criteria, including 10 randomized controlled trials and 15 observational studies, totaling 6430 patients. The RESCUEicp trial (n = 408) found that decompressive craniectomy for late refractory ICP reduced mortality (26.9% vs. 48.9% in medical management) but increased severe disability rates [8]. The DECRA trial (n = 155) showed that early decompressive craniectomy reduced ICP and ICU stay but did not improve functional outcomes, with higher disability rates compared with medical management [9]. Guideline updates in 2020 incorporated these findings, emphasizing large craniectomies and selective use of surgery [7]. Non-surgical management, including osmotherapy and sedation, effectively controlled ICP in 60% of cases across studies [10]. However, access to advanced monitoring (e.g., invasive ICP devices) varied, particularly in low-resource settings, impacting outcomes [11].

### 3.2. Pathophysiology

TBI begins with a primary insult from mechanical forces, such as blunt trauma, falls, blast injuries, or sudden cranial velocity shifts, leading to injury patterns like focal contusions, diffuse axonal injury (DAI) from shear forces, and intracranial hemorrhages [2,12]. DAI most commonly occurs from high-speed motor vehicle accidents, during which sudden deceleration causes shearing forces that disrupt the axons at the gray and white matter junction. DAI is most commonly classified using the Adams Diffuse Axonal Injury grading system, ranging from Grade 1 (most mild) to Grade 3 (most severe). The severity of the primary injury and initial clinical presentation guide management decisions. For example, focal injuries may require surgical evacuation, while DAI, characterized by widespread axonal disruption, often necessitates medical management [13]. Neuroimaging, particularly computed tomography (CT) and magnetic resonance imaging (MRI), correlates injury patterns with clinical severity, aiding prognosis [14].

Secondary injury, occurring within minutes to days, involves a complex cascade of molecular and systemic processes. Reduced cerebral blood flow from thrombosis, vasospasm, or hypotension leads to hypoxia or ischemia [12]. Intracellular calcium surges disrupt mitochondrial function, triggering excitotoxicity and neuronal death [15]. Cytokines and free radicals drive oxidative stress and inflammation, exacerbating tissue damage and blood–brain barrier dysfunction [16]. These processes increase cerebral edema, elevating intracranial pressure (ICP) and reducing cerebral perfusion pressure (CPP), calculated as mean arterial pressure (MAP) minus ICP [17]. The Monro–Kellie doctrine, proposed in 1783 and confirmed in 1824, describes the cranium as a rigid box containing brain, blood, and cerebrospinal fluid (CSF), where an increase in one component (e.g., edema) raises ICP, compromising CPP [17]. Systemic effects, such as autonomic dysfunction (e.g., paroxysmal sympathetic hyperactivity), further complicate management by causing hemodynamic instability [18]. Emerging research highlights the role of neuroinflammation in long-term neurodegeneration, potentially linking TBI to chronic traumatic encephalopathy [19]. This critical window of secondary injury underscores the need for timely interventions to halt the destructive cycle of excitotoxicity, inflammation, and necrosis [20].

### 3.3. Management

Effective TBI management requires a multidisciplinary approach, encompassing prehospital care, acute interventions, surgical and non-surgical strategies, intensive care monitoring, and long-term rehabilitation. The primary goals are to stabilize the patient, mitigate secondary injury, and optimize neurological recovery, guided by evidence-based protocols such as the Brain Trauma Foundation’s 2020 guidelines [7,10]. Management of TBI often involves consultations with neurosurgery, neurology, trauma surgery, and intensive care specialists, who together guide therapies.

Furthermore, TBI often affects not only patients, but their families as well. Patients with TBI, and specifically high-grade DAI, routinely experience a wide range of neurologic deficits and mental status changes. Because recovery from severe TBI can take several months to multiple years, consideration of family support must be given. This is particularly important because TBI impacts pediatric patients at a greater rate than many other neurologic conditions.

### 3.4. Prehospital Care

Prehospital management follows Advanced Trauma Life Support (ATLS) guidelines, prioritizing airway protection, oxygenation, and hemodynamic stabilization [10,21]. Securing the airway is critical for patients with a Glasgow Coma Scale (GCS) score of <8 or significant facial trauma, risking airway obstruction or aspiration [21]. Hypoxia, a key contributor to secondary injury, is prevented through supplemental oxygen or mechanical ventilation to maintain oxygen saturation above 90% [22]. Emerging research suggests that hypoxia and hypotension are associated with increased mortality in patients with TBI. Hypotension (systolic blood pressure < 90 mmHg) exacerbates cerebral ischemia and is addressed with isotonic to hypertonic fluids, avoiding hypotonic solutions that may worsen edema [10]. Cervical spine immobilization is standard to prevent exacerbating occult injuries. Rapid transport to a trauma center with neurosurgical capabilities is essential, as delays in treatment increase mortality [23].

### 3.5. Acute Interventions

Upon hospital arrival, a primary survey to assess airway, breathing, and circulation should be performed, followed by a secondary neurological examination to determine GCS and pupillary responses [10]. Following a thorough neurological examination, neuroimaging should be obtained to assess the severity of the TBI. Non-contrast computed tomography (CT) is the cornerstone of neuroimaging, identifying hematomas, contusions, and skull fractures that guide management decisions [14]. For example, epidural or subdural hematomas with significant mass effect often require urgent surgical evacuation [24]. Depending on the mechanism of injury and suspicion of vascular injury, CT angiogram (CTA), the decision to order a CTA of the neck is guided by the use of the Denver criteria following trauma. Classically, high-energy transfer mechanisms of injury, risk factors, and signs and symptoms of cerebrovascular injury are the criteria used in decision-making for patient selection for CTA of the neck and head [25].

Principles of acute interventions rely primarily on managing ICP. ICP management is approached using a tiered system. Firstly, the head of the bed should be elevated to 30 degrees, hyponatremia should be avoided, analgesics should be administered to reduce pain, and the head should be midline with appropriate fit of cervical collar if applicable. Secondly, medications can be administered to decrease ICP. Initial medical management includes administering mannitol or hypertonic saline for cerebral edema and using anticonvulsants (e.g., levetiracetam) to prevent early post-traumatic seizures [26]. Sedation with propofol or midazolam may be employed to control agitation and reduce metabolic demand, though prolonged use requires monitoring for complications like propofol infusion syndrome [10].

### 3.6. Surgical Management

Surgical intervention is indicated for mass lesions (e.g., hematomas) causing neurological deterioration or refractory ICP elevation [7]. Hematoma evacuation via craniotomy is prioritized for epidural hematomas or large subdural hematomas with a midline shift of >5 mm [24]. Decompressive craniectomy, the removal of a portion of the skull to alleviate ICP, has been rigorously evaluated in trials like RESCUEicp and DECRA [8,9]. The Brain Trauma Foundation’s 2020 guidelines, updated post-RESCUEicp, provide four level-IIA recommendations: (1) decompressive craniectomy for late refractory ICP (>24 h) improves mortality and functional outcomes; (2) early decompressive craniectomy (<24 h) for refractory ICP is not recommended for improved outcomes, distinct from primary craniectomy for mass lesions; (3) large frontotemporoparietal craniectomies (≥12 × 15 cm) are preferred over smaller ones for better mortality and neurological outcomes; and (4) secondary decompressive craniectomy reduces ICP and ICU stay, though its effect on long-term outcomes remains uncertain [7]. The RESCUE-ASDH trial further clarified that primary decompressive craniectomy for acute subdural hematoma offers no clear advantage over craniotomy in terms of functional outcomes, highlighting the need for individualized surgical decisions [27].

### 3.7. Intensive Care Unit Management

In the ICU, the focus shifts to preventing secondary injury through precise monitoring and management of ICP and CPP [10]. Invasive ICP monitoring, typically via an intraventricular catheter or intraparenchymal probe, is recommended for patients with GCS < 8 and abnormal CT findings [7]. Target ICP is <22 mmHg, with CPP maintained between 60 and 70 mmHg, achieved through vasopressors (e.g., norepinephrine) or CSF drainage [28]. Advanced neuromonitoring, such as brain tissue oxygen (PbtO_2_) monitoring or cerebral microdialysis, provides insights into tissue oxygenation and metabolic status; however, availability is limited to specialized centers, and improvements in outcomes from these measures need further investigation [29]. Non-surgical interventions include osmotherapy (mannitol or hypertonic saline) for acute ICP spikes, controlled hyperventilation (PaCO_2_ 32–35 mmHg) for short-term ICP control, and barbiturate coma for refractory cases, despite risks of hypotension [10]. Targeted temperature management (normothermia, 36–37 °C) is preferred over therapeutic hypothermia, following the EUROTHERM3235 trial’s findings of no outcome benefit [30]. Frequent neurological assessments and serial CT scans guide therapy adjustments, with multidisciplinary input from neurosurgeons, intensivists, and neurologists [23].

### 3.8. Rehabilitation and Long-Term Care

Post-acute management focuses on rehabilitation to address physical, cognitive, and psychological deficits. Early rehabilitation, initiated in the ICU, includes physical therapy to prevent contractures and occupational therapy to restore daily functioning [31]. Cognitive rehabilitation targets memory, attention, and executive function deficits, often using computer-based training or compensatory strategies [31]. Psychological support, including cognitive behavioral therapy, addresses post-traumatic stress disorder and depression, which affect up to 30% of TBI survivors [32]. Long-term care may involve outpatient follow-up, vocational rehabilitation, and community reintegration programs to optimize quality of life [31]. Emerging neuroprotective agents, such as erythropoietin or statins, are under investigation but lack robust evidence for routine use [20]. It is thought that erythropoietin may reduce rates of apoptosis, decrease inflammation, and contribute to increased cerebral blood flow. Despite these proposed mechanisms of action, data thus far have been unconvincing regarding their effectiveness. Similarly, it is thought that statins decrease inflammation and enhance cerebral blood flow, although data thus far have also been unconvincing.

Furthermore, patients with TBI can develop seizure disorder and epilepsy. In fact, post-traumatic epilepsy accounts for 10–20% of epilepsy cases in the general population, and over half of epilepsy cases in veterans [33]. Although the latency period for seizures following TBI is variable, around 80% of patients who develop post-traumatic epilepsy experience their first seizure within the first year following their initial injury, and 90% within the first 2 years following TBI. Development of post-traumatic epilepsy correlates most with the severity of TBI, with more severe cases being more likely to develop the condition [34].

### 3.9. Guideline Updates and Future Directions

The Brain Trauma Foundation’s 2020 guidelines, incorporating 28 recommendations, emphasize evidence-based ICP management and selective surgical intervention [7]. The Congress of Neurological Surgeons’ “living guidelines” initiative ensures timely updates, reflecting trials like RESCUEicp and RESCUE-ASDH [8,27]. Future management strategies may leverage precision medicine, using biomarkers (e.g., S100B, GFAP) to tailor therapies, and wearable technologies for real-time monitoring in low-resource settings [29]. Addressing global disparities in access to neurosurgical care and advanced monitoring remains a priority to improve TBI outcomes worldwide [11]. The complete guidelines from the Brain Trauma Foundation can be found at the following link: https://braintrauma.org/coma/guidelines-current (accessed on 20 July 2025).

## 4. Discussion

The evolving landscape of TBI management reflects the interplay between rigorous clinical trials and practical challenges in implementation. The RESCUEicp trial demonstrated that decompressive craniectomy for late refractory ICP significantly reduces mortality, offering a lifeline for patients with severe TBI [8]. However, the increased incidence of severe disability raises ethical questions about the quality of life for survivors, necessitating careful patient selection and family counseling [35]. In contrast, the DECRA trial’s findings caution against early decompressive craniectomy for refractory ICP, as it fails to improve functional outcomes and may increase harm [9]. These results echo earlier concerns about overly aggressive surgical interventions, as seen in trials like the STITCH(Trauma) study, which found no clear benefit for early hematoma evacuation in certain cases [24]. The Brain Trauma Foundation’s 2020 guidelines strike a balance, advocating for large craniectomies in specific scenarios while emphasizing non-surgical strategies like osmotherapy and sedation [7,10]. Certainly, appropriate patient selection is essential when deciding whether surgical intervention is appropriate for a patient with severe TBI. It is accepted that medical management is the first step in the treatment of TBI; however, when TBI is refractory to medical therapy, decompressive craniotomy should be considered, as supported by the latest research.

The shift to “living guidelines” by the Congress of Neurological Surgeons is a forward-thinking response to the rapid pace of TBI research, allowing real-time updates to reflect trials like RESCUE-ASDH, which explored primary decompressive craniectomy in acute subdural hematoma [27]. However, controversies persist, particularly around the optimal timing and indications for surgery. For instance, some experts argue that RESCUEicp’s focus on late ICP elevation overlooks patients with mixed injury patterns, where earlier intervention might be beneficial [36]. Non-surgical advances, such as targeted temperature management and neuroprotective agents, show promise but lack definitive evidence, as seen in the disappointing results of the EUROTHERM3235 trial [30]. Global disparities in TBI care further complicate guideline implementation. In low- and middle-income countries, where 90% of TBI deaths occur, access to neurosurgical expertise and ICP monitoring is limited, leading to worse outcomes [11]. Rural hospitals in high-income countries face similar challenges, often requiring patient transfers that delay critical interventions [37].

While there are interventions for controlling ICP, the subset of patients with severe TBI who also have DAI continue to experience bad outcomes, particularly if their DAI is grade two or three. In fact, the mortality rate of patients with DAI has been reported to be as high as 42–62% [38]. Because the mechanism of DAI involves shearing forces that sever the nerve fibers and cause secondary insult, it is difficult to treat effectively. Treatment for patients with DAI continues to evolve. At present, there are few effective therapies besides intensive rehabilitation for these patients.

Emerging research into TBI pathophysiology offers hope for novel therapies. For example, studies on neuroinflammation suggest that anti-inflammatory agents could mitigate secondary injury, though clinical translation remains elusive [19]. Similarly, advances in neuromonitoring, such as microdialysis and brain tissue oxygen sensors, may further enable personalized treatment strategies [29]. However, these technologies are costly and unavailable in many settings, highlighting the need for scalable solutions. The limitations of this review include the heterogeneity of included studies, potential publication bias, and a focus on English-language literature, which may exclude valuable global perspectives. Future research should prioritize long-term outcome studies, cost-effective monitoring tools, and access to care.

## 5. Conclusions

TBI remains a global health challenge, with management strategies evolving through rigorous clinical trials and guideline updates. The 2020 Brain Trauma Foundation guidelines, informed by RESCUEicp and DECRA, provide evidence-based recommendations for decompressive craniectomy and ICP management, balancing mortality benefits with functional outcomes. Non-surgical interventions and vigilant monitoring are equally vital, particularly in resource-constrained settings. The move toward “living guidelines” reflects the dynamic nature of TBI research, ensuring timely integration of new evidence. Future efforts should focus on improving access to advanced care, standardizing protocols, and evaluating long-term outcomes to enhance the quality of life for TBI patients.

## Data Availability

All data included in this study can be found in the reference section. No new data sets were created for this study.

## Abbreviations

The following abbreviations are used in this manuscript:

ATLS	Advanced Trauma Life Support
CPP	Cerebral Perfusion Pressure
CSF	Cerebrospinal Fluid
CT	Computed Tomography
CTA	Computed Tomography Angiography
DAI	Diffuse Axonal Injury
GCS	Glasgow Coma Scale
GFAP	Glial Fibrillary Acidic Protein
ICP	Intracranial Pressure
MAP	Mean Arterial Pressure
MRI	Magnetic Resonance Imaging
PaCO_2_	Partial Pressure of Carbon Dioxide
PbtO_2_	Brain Tissue Oxygen Partial Pressure
TBI	Traumatic Brain Injury
